# Correction: Terephthalate and trimesate metal–organic frameworks of Mn, Co, and Ni: exploring photostability by spectroscopy

**DOI:** 10.1039/d1ra90131a

**Published:** 2021-07-22

**Authors:** Nishesh Kumar Gupta, Jiyeol Bae, Suho Kim, Kwang Soo Kim

**Affiliations:** University of Science and Technology (UST) Daejeon Republic of Korea; Department of Land, Water, and Environment Research, Korea Institute of Civil Engineering and Building Technology (KICT) Goyang Republic of Korea kskim@kict.re.kr

## Abstract

Correction for ‘Terephthalate and trimesate metal–organic frameworks of Mn, Co, and Ni: exploring photostability by spectroscopy’ by Nishesh Kumar Gupta *et al.*, *RSC Adv.*, 2021, **11**, 8951–8962, DOI: 10.1039/D1RA00181G.

The authors regret that an incorrect grant number was given in the Acknowledgements section of the original article.

The corrected acknowledgments are as follows:

The authors are very grateful for the funds [Project #20210152-001] provided by the “Korea Institute of Civil Engineering and Building Technology” (KICT), Republic of Korea.

The Royal Society of Chemistry apologises for these errors and any consequent inconvenience to authors and readers.

## Supplementary Material

